# A Case of Neurofibroma in the Supraumbilical Region Undergoing Cystic Myxomatous Degeneration: An Unusual Presentation

**DOI:** 10.7759/cureus.54246

**Published:** 2024-02-15

**Authors:** Yogesh B Manek, Suhas Jajoo, Chandrashekhar Mahakalkar, Shivani Kshirsagar

**Affiliations:** 1 General Surgery, Jawaharlal Nehru Medical College, Datta Meghe Institute of Higher Education and Research, Wardha, IND

**Keywords:** degeneration, myxomatous, swelling, primary umbilical neoplasms, neurofibroma

## Abstract

Primary umbilical neoplasms are exceptionally rare. Neurofibromas histologic findings vary from collagenous to myxoid matrix according to the neoplastic elements differentiation. We present a case of neurofibroma in the supraumbilical region undergoing cystic myxomatous degeneration. A 75-year-old female presented to the department with a complaint of swelling above the umbilicus for the last three years. The swelling was excised and grossly was a single, irregular, reddish-yellow cystic mass measuring 4.5 × 4 x 3 cm. On the cut section, cystic jelly-like areas were identified, and histopathological features were suggestive of neurofibroma undergoing cystic myxomatous degeneration. The diagnosis needed to be combined with pathological examination, and careful consideration during the surgical intervention was important to confirm there was no residue.

## Introduction

A benign neuromatoid proliferative lesion that originates from the peripheral nerve sheath, specifically the endoneurium, is called neurofibroma [1]. Neurofibromas typically start as neurites, Schwann cells, or fibroblasts, among other cells that make up the nerve sheath. Nevertheless, it is unclear which specific cell causes a neurofibroma to develop [2]. It may be a solitary lesion or a component of a neurofibromatosis. Among the variations of solitary neurofibroma that may manifest are the cutaneous lipomatous, collagenous, epithelioid, granular, pigmented, dendritic cells, and myxoid neurofibromas [3]. The onset age is between 20 and 40 years, and there is no discernible difference between males and females. The histologic findings of neurofibromas differ based on the differentiation of the neoplastic components, ranging from collagenous to myxoid matrix [2].

## Case presentation

**Figure 1:**
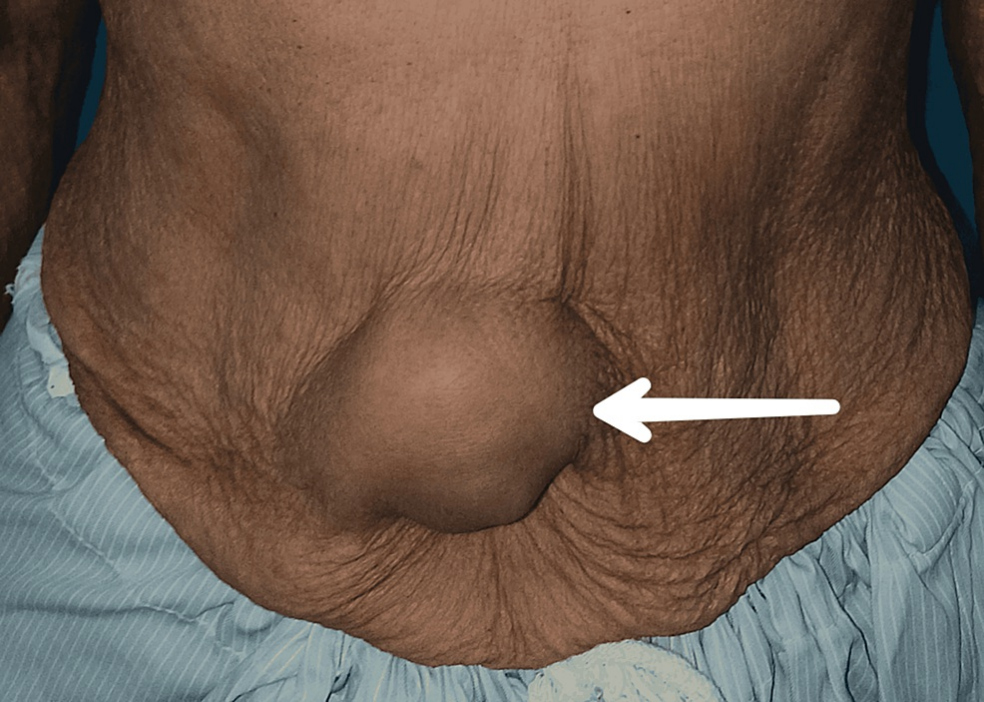
Clinical picture of swelling over the supraumbilical region Supraumbilical swelling of approximately 4 x 4 cm in size, oval in shape, smooth in surface, firm in consistency, non-reducible, and mobile

A 75-year-old female presented to the Department of Surgery with a complaint of swelling above the umbilicus for the last three years. The patient was alright three years back when she started having swelling over the supraumbilical region (Figure 1). Initially, it was 1 x 1 cm and currently 4 x 4 cm, and associated with a dull aching pain, which was insidious in onset, gradually progressive, and non-radiating.

**Figure 2:**
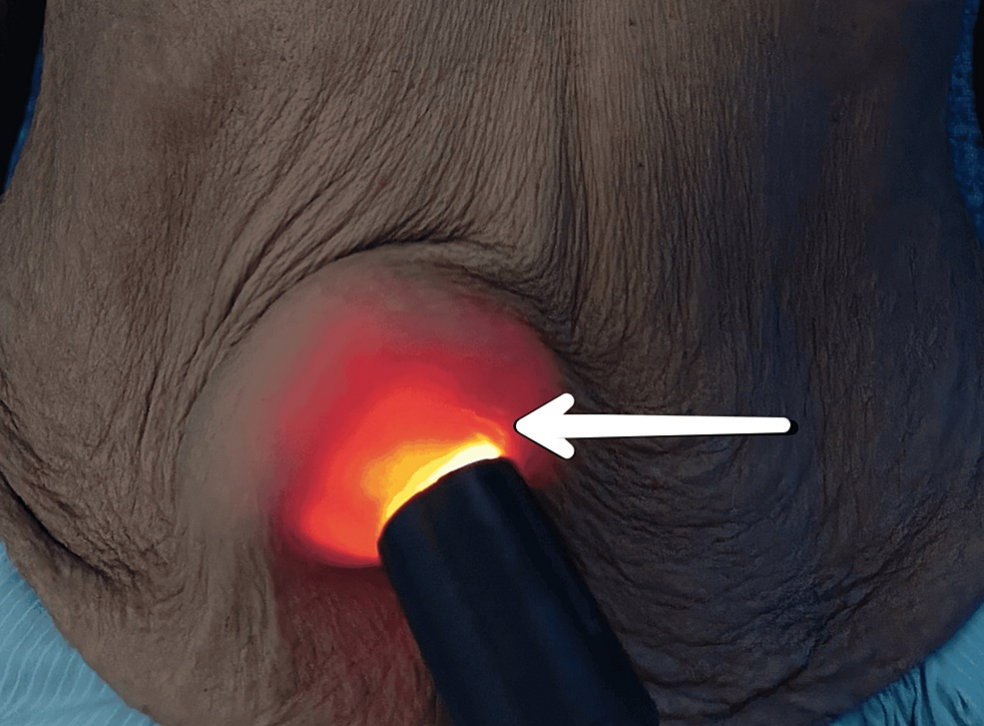
Positive transillumination test A cystic swelling above the umbilicus displaying vivid transillumination indicates its cyst-like characteristics.

The medical history reported nothing notable except the patient's well-controlled hypertension, and she had no positive family history of a similar disease. There was no other swelling in the body and no previous history of surgical intervention. A local examination revealed a single swelling of size 4 x 4 cm in the supraumbilical region. The swelling had a smooth surface, and hyperpigmentation was present over it. It was firm in consistency, and no local temperature rise was present. The fluctuation and transillumination tests were positive, suggesting clean fluid containing cystic swelling (Figure 2).

**Figure 3:**
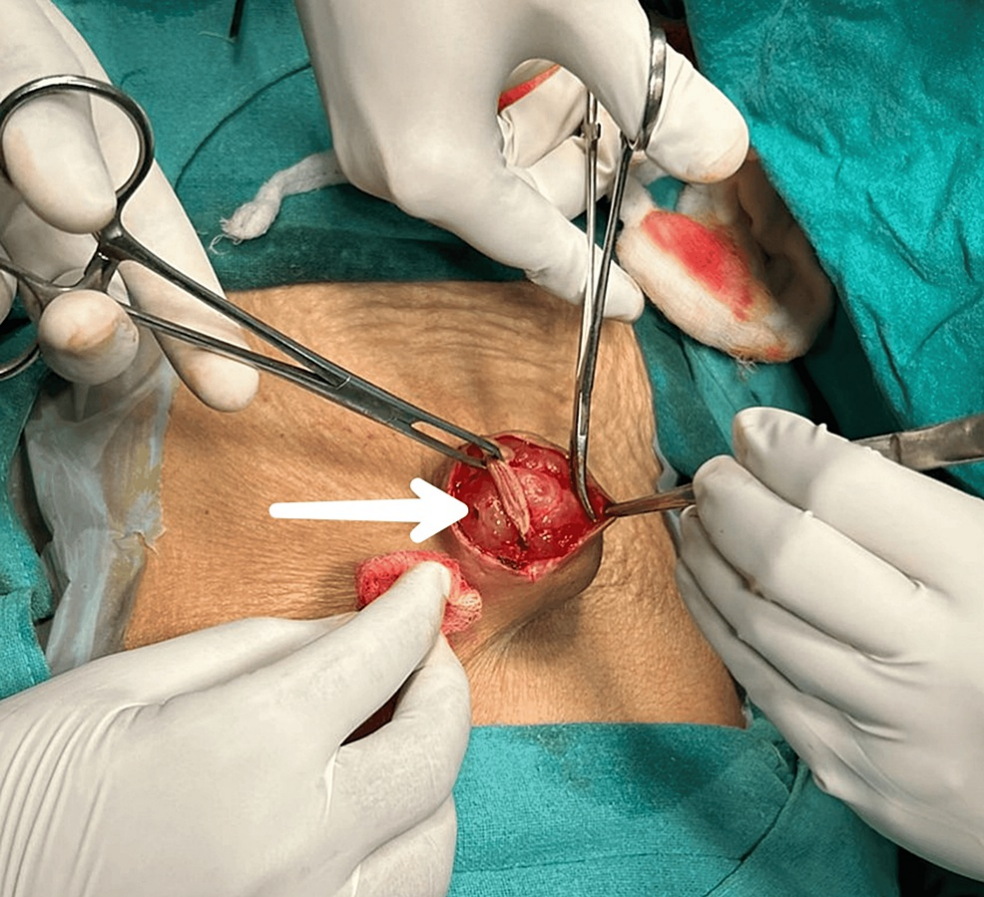
Intraoperative picture of excision of supraumbilical swelling

The swelling was mobile, non-reducible, non-compressible, and fixed to the skin but not fixed to underlying tissues. No pulsations were present, and cough impulses were negative. Systemic examination revealed no abnormality at any other site. The provisional diagnosis was a well-defined cyst, lymphatic and protruding from the supraumbilical region. An ultrasound was done and the findings were suggestive of a heterogeneous collection or lesion with vascularity on Doppler, indicating a possible cystic nature. The differential diagnosis was hemangioma, hernia, benign or malignant cutaneous tumor, or metastatic tumor. A fine-needle aspiration cytology (FNAC) was recommended to further diagnose the condition. However, FNAC was not performed due to the risk of swelling rupture before surgery. Further cross-sectional imaging was not feasible due to financial constraints on the part of the patient. It was excised completely and sent for histopathological examination (Figure 3).

**Figure 4:**
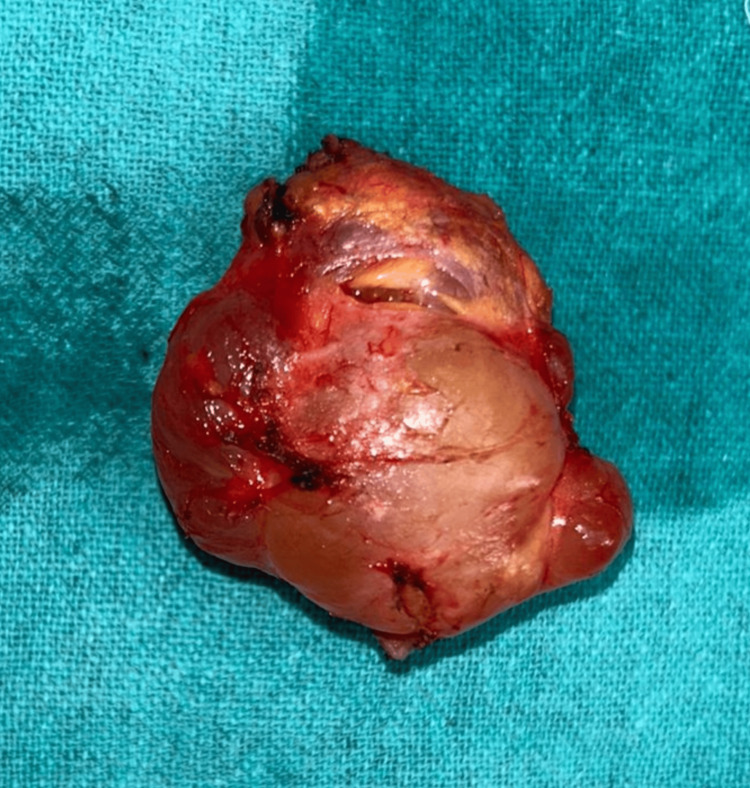
Excisional biopsy. Gross examination shows a single, irregular, reddish-yellow cystic mass measuring 4.5 x 4 x 3 cm.

Grossly, the swelling was a single, irregular, reddish-yellow cystic mass measuring 4.5 × 4 x 3 cm (Figure 4).

**Figure 5:**
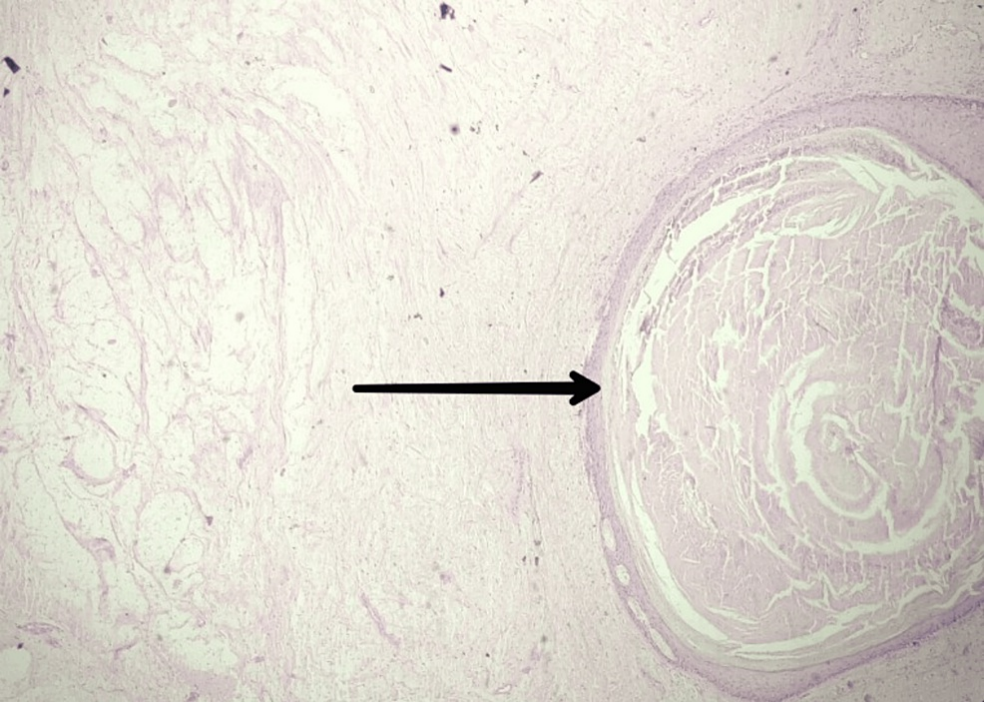
Section shows a sparse number of Schwann cells scattered in a myxoid stroma with vascularity (H&E 4x). The specimen contains diverse amounts of mucinous stroma, collagen, and cellular elements. H&E: Hematoxylin and eosin

On the cut section, cystic jelly-like areas were identified, and histopathological features were suggestive of neurofibroma undergoing cystic myxomatous degeneration (Figure 5).

At the time of discharge, there were no indications of wound infection or wound dehiscence, and the scar was deemed satisfactory. The postoperative period proceeded without any complications, and there were no instances of recurrence or hypertrophic scarring during the follow-up period.

## Discussion

One of the most frequent benign tumors of the peripheral nerve, neurofibroma, was initially described by Verocay in 1910. It is currently thought that Schwann cells in the nerve sheath are the source of neurofibromas. Ninety percent of neurofibromas have a unilateral onset and can affect any area of the body. They are more common in young and middle-aged individuals [1,4]. Myxomatous neurofibromas are most frequently found in the face, shoulders, arms, periungual area, and foot [3].

This is the first case of a neurofibroma of the supraumbilical region location that we are aware of. The umbilicus is regarded by doctors as a crucial component in the diagnosis of specific illnesses. The umbilicus is made up of the remaining portions of the umbilical cord and its adnexa, which are only visible during pregnancy. Since the umbilicus connects with the mentioned organs through these channels, the majority of the venous and lymphatic drainage of numerous solid internal organs goes through this region, making any odd alterations to this area highly relevant in the eyes of medical professionals [5].

The umbilicus is a unique structure with several embryonic remnants and a sophisticated braided lymphatic network drainage system. It is not merely a section of skin that can be affected by other skin neoplasms like any other region of the body [6]. Thus, malignant tumors originating from internal solid organs frequently move through the umbilicus. As internal organ cancers spread, skin metastases in the umbilicus, whose primary cancers are typically found in the pelvic or abdominal region called "Sister Mary Joseph nodules" in honor of Sister Mary Joseph Dempsey, the surgical assistant who first introduced Dr. Mayo, the founder of the Mayo Clinic, to the idea of metastatic umbilical tumors [5]. In addition to these cancerous diseases, nevi, teratomas, papillomas, and a case of cutaneous ciliated cyst have all been recorded as benign neoplasms in the umbilicus. Imaging tests were conducted to rule out a relationship between the mass and intraabdominal organs after this benign cyst aroused concerns about the possibility of an embryonic remnant [7]. The condition can be observed in pregnant women and after abortion [8-10].

In this case, an examination of the abdomen, musculoskeletal system, and gynecological system revealed no abnormalities. Like in our patient, myxomatous neurofibromas are typically solitary lesions, although they can also be many and may return after an imperfect first excision [3]. It is possible to do a transillumination test to ascertain if a mass is solid (light blocked by the mass) or cystic (light shines through) by holding an external light against the umbilicus [11]. In this instance, light shone through, meaning that a positive transillumination test suggested the presence of clear fluid with cystic enlargement. Additionally, a well-defined cyst from the supraumbilical region was the indicative preliminary diagnosis.

Histologically, Schwann cells, perineural cells, endoneurial fibroblasts, and intermediate cells make up solitary neurofibromas, which come from the nerves. Their distinct boundaries with the surrounding connective tissue set them apart from the numerous neurofibromas seen in neurofibromatosis instances. Immunostaining biopsy samples with S-100 and CD34 is essential. S-100 protein-positive tumor cells are less numerous in neurofibromas than in schwannomas, and the tumor cells are loosely organized and fragile, frequently with wavy or snake-shaped nuclei [12]. Hemangioma, hernia, benign or malignant cutaneous tumor, and metastatic tumor were the differential diagnoses.

Therefore, FNAC was not performed since it could rupture the edema and cause the cyst to leak before surgery. In this instance, the bulge was removed and sent for histological analysis. Measuring 4.5 × 4 x 3 cm, it was an uneven, solitary, reddish-yellow cystic tumor. Cystic jelly-like material was found on the part dissected, and the histological characteristics pointed to neurofibroma that was going through cystic myxomatous degeneration. The diagnosis can be made more easily if the age of manifestation and distinctive clinical characteristics of umbilical lesions are known. Although FNAC can also be tried, histopathology is still the best option in questionable circumstances. Because histopathological assessment is important for both diagnosis and prognosis in umbilical lesions, it is required. Particularly in adults, any umbilical growth or nodule should be carefully examined since it may be the lone indicator of an underlying, hidden cancer [13].

## Conclusions

Here, we presented a case with an unusual presentation of neurofibroma in the supraumbilical region undergoing cystic myxomatous degeneration. The diagnosis needed to be combined with pathological examination, and careful consideration during the surgical intervention was important to confirm that there was no residue. The complete resection of pathology is pivotal to prevent recurrence. The umbilical lesions required meticulous diagnosis as these lesions may reflect hidden primary disorder or malignancy. Detecting such lesions at the earliest is crucial for preventing complications. While some necessitate emergent intervention, others can be managed conservatively.
